# Pig Abattoir Inspection Data: Can It Be Used for Surveillance Purposes?

**DOI:** 10.1371/journal.pone.0161990

**Published:** 2016-08-26

**Authors:** Carla Correia-Gomes, Richard P. Smith, Jude I. Eze, Madeleine K. Henry, George J. Gunn, Susanna Williamson, Sue C. Tongue

**Affiliations:** 1 Epidemiology Research Unit, Future Farming Systems Research Group, Scotland’s Rural College, Kings Building, West Mains Road, Edinburgh, United Kingdom; 2 Animal and Plant Health Agency, New Haw, Addlestone, Weybridge, Surrey, United Kingdom; 3 Animal and Plant Health Agency, Rougham Hill, Bury St Edmunds, Suffolk, United Kingdom; Charles University in Prague, CZECH REPUBLIC

## Abstract

Statutory recording of carcass lesions at the abattoir may have significant potential as a resource for surveillance of livestock populations. Food Standards Agency (FSA) data in Great Britain are not currently used for surveillance purposes. There are concerns that the sensitivity of detection, combined with other issues, may make the outputs unreliable. In this study we postulate that FSA data could be used for surveillance purposes. To test this we compared FSA data with BPHS (a targeted surveillance system of slaughtered pigs) and laboratory diagnostic scanning surveillance (FarmFile) data, from mid-2008 to mid-2012, for respiratory conditions and tail bite lesions in pigs at population level. We also evaluated the agreement/correlation at batch level between FSA and BPHS inspections in four field trials during 2013. Temporal trends and regional differences at population level were described and compared using logistic regression models. Population temporal analysis showed an increase in respiratory disease in all datasets but with regional differences. For tail bite, the temporal trend and monthly patterns were completely different between the datasets. The field trials were run in three abattoirs and included 322 batches. Pearson’s correlation and Cohen’s kappa tests were used to assess correlation/agreement between inspections systems. It was moderate to strong for high prevalence conditions but slight for low prevalence conditions. We conclude that there is potential to use FSA data as a component of a surveillance system to monitor temporal trends and regional differences of chosen indicators at population level. At producer level and for low prevalence conditions it needs further improvement. Overall a number of issues still need to be addressed in order to provide the pig industry with the confidence to base their decisions on these FSA inspection data. Similar conclusions, at national level, may apply to other livestock sectors but require further evaluation of the inspection and data collection processes.

## Introduction

Endemic diseases are a particular concern to all livestock industry sectors, causing production losses and welfare issues. Surveillance can play an important role in their control by the provision of estimates of the frequency, such as prevalence or incidence, of disease and/or welfare conditions. These estimates can be monitored over time and significant changes detected [1]. Detection of change may lead to the initiation of appropriate interventions with continued surveillance enabling the evaluation of their impact. The units of analysis may vary from the population level to the individual animal and the outputs may be similarly targeted; from population (industry/government/region) to an individual producer level. Each purpose, or surveillance objective, may ideally need the data collection and/or analysis to be designed differently to produce the required output [2,3]. Asking a surveillance system to meet multiple objectives will require multiple components and, potentially, compromises will need to be made. When resources are limited, cost effective surveillance methods are also important; hence, existing data sources are being explored to determine if they can be used for surveillance purposes [4,5,6,7].

In Great Britain, probably the most robust data sources [8] that are currently used, or have the potential to be used, for endemic disease surveillance in livestock species are the Animal and Plant Health Agency (APHA)’s FarmFile system and statutorily collected abattoir inspection data [8]. Furthermore the pig sector also has robust health schemes [9], which were purposively designed for specific surveillance purposes. S1 Table describes these three systems in detail for pigs. Briefly abattoir inspection started in the late 1800s [10], with the objective to identify and discard carcasses infected with major zoonotic pathogens and parasitic infections in order to reduce public health risks. Since then, abattoir inspection has expanded and now has several purposes associated with public health, animal health and welfare and meat quality. In Great Britain, abattoir inspection has been audited and controlled by the Food Standards Agency (FSA). It is performed by the official auxiliaries (commonly known as meat hygiene inspectors (MHIs)) and official veterinarians. The data for the conditions/lesions observed and recorded can be provided to the farmer and the farmer’s private veterinary surgeon, to allow them to take action on-farm to improve animal health and welfare i.e. it has the potential to be used at a producer level. However, the abattoir inspection data are not currently being used for surveillance purposes at population level, e.g. to monitor trends of specific conditions or to detect significant changes in endemic disease trends. The pig health schemes in Great Britain were designed to provide information about specific endemic conditions of economic concern for producers, with relevant outputs provided both to individual producers and at a population (industry) level [11]. In Great Britain there are two schemes: Wholesome Pigs Scotland, in Scotland, and the British Pig Health Scheme (BPHS), in England and Wales. BPHS started in 2005 and provides frequent feedback of benchmarked results from targeted abattoir inspections to the participating producers and their herd veterinarians [8], helping to increase their awareness of the occurrence of subclinical diseases in their farms [11]. Since 1999, farm and laboratory data collected from voluntary laboratory submissions made across Great Britain, to APHA Regional Laboratories and Scottish Agricultural College’s Consultancy Division’s Disease Surveillance Centres has been aggregated in the FarmFile database [12]. Carcass and non-carcass submissions are submitted through private veterinary surgeons for laboratory testing and diagnostic investigation. Individual results are sent back to the private veterinary surgeon and the data contribute to surveillance for the detection of new and emerging threats, including significant changes in endemic disease trends.

Denmark and the Netherlands have pioneered the use of abattoir inspection data as a means of animal health surveillance, followed by countries such as Sweden, Norway and Germany [10]. Advantages include: increased coverage using an existing data collection system and data on many health and welfare conditions. Such data could be used to help identify potential emerging animal health threats, or to target slaughtered animals entering the food chain that present a direct risk to people from zoonotic organisms. However, difficulties have been identified with FSA data in the past and there is a lack of trust in the system. Some of the issues that have been identified with the FSA include a lack of sensitivity (ability to detect an affected carcass); a lack of standardisation with multiple operators and conditions; poor data quality and coverage of only those healthy pigs sent to slaughter [13].

The aim of this study was to test if abattoir inspection data has potential for surveillance purposes: firstly at a population level, to measure how much disease is present? and how this changes over time, and secondly at a producer level, to provide information to direct individual action, using the pig industry and respiratory conditions and tail bite lesions, as an example. Respiratory conditions were chosen as they are one of the major diseases affecting pigs worldwide [14]. Most of the respiratory lesions in pigs in Great Britain are lung consolidation likely to be caused by enzootic pneumonia (EP) and pleurisy [9]. Tail bite lesions are considered a welfare problem, especially in Great Britain, where routine tail docking of pigs is banned. These lesions may indicate stress, adverse environmental conditions and intercurrent disease among other factors [15].

To achieve this aim the FSA data were compared with data from the two existing data sources commonly used to monitor trends in endemic disease: FarmFile and BPHS. The specific objectives were a) to compare the prevalence (over the time period and spatial distribution) of FSA data with BPHS and FarmFile for respiratory conditions and tail bite lesions, looking for similarities and differences, and b) to examine the agreement between BPHS and FSA data–the question here being, could FSA replace BPHS data?

## Materials and Methods

### Population level comparison

#### Data description

The data sources used for the population level analysis were FSA, BPHS and FarmFile data. The details of these systems and type of data collected are explained in detail in S1 Table. Datasets were collected to cover a 48 month period from June 2008 to May 2012. However, the FSA dataset only started at August 2009, following the implementation of a new electronic format for the data.

#### Data management and analyses

To assess regional prevalences, the postcode of the farm of origin of the carcass, or submission, was used to link a record to a geographical region, based on British NUTS 1 regions (Nomenclature of Units for Territorial Statistics [16]). Where full postcodes were missing, county definitions were used to classify which region the record belonged to. The FSA dataset did not include postcode information, so other information available to the researchers was used to link a postcode to each herd/slap mark in the dataset. Slap mark is the herd mark that is tattooed on a pig and identifies farm of origin. It is a legally required official reference for each pig farm. In some cases a farm can have more than one slap mark. It was not possible to identify those cases in our analysis so it was assumed that each slap mark will approximate to a farm.

Two conditions were considered: respiratory conditions and tail bite lesions. As each data source has different ways of assessing some of the same lesions/diseases, the data were recategorised into “lesions/diseases” (S2 Table). The FSA data include two inspection types: ante mortem (AM) and post mortem (PM). Within the PM inspection, conditions were recorded as present in the carcass, offal, or whether they were generalised conditions.

For each of the datasets, the date of assessment was used to produce variables for the year and month of assessment. Regional prevalences were calculated and plotted in a map. Epidemiological modelling was used to detect statistically significant differences (P<0.05) between the results. The data from each data source was modelled separately.

#### Respiratory lesions

All three datasets were used for the analysis of respiratory conditions. In the FSA data tuberculosis (TB)-like lesions and suspected generalised tuberculosis were included, although it should be noted that these are not necessarily respiratory conditions. For the FarmFile system interpretation was required to decipher multiple diagnoses from the same submission to clarify if a respiratory condition was present. A submission may relate to multiple animals being ill on a holding with samples being collected from single or multiple pigs, but typically a diagnosis for a single pig is produced and so for the purposes of this analysis we have assumed that a submission is related to a single pig with a diagnosed condition. For the FSA and BPHS dataset the number of animals assessed and the number of individual animals that had any positive result for any of the respiratory conditions were summarised per batch. For the FarmFile dataset a binary score was created for each submission, based on the presence/absence of any respiratory conditions, and the results summarised per submission.

As each of the data sources contained multiple samples from the same farm, this contravenes the assumption of independence between records. Generalised linear mixed models (GLMM) were used to account for this. The unique farm identifier (slap mark) recorded in each data source was applied as a random effect in the GLMM to account for the potential similarity of results between samples from the same farm. GLMM also adequately corrects for the differences in sample size between batches by incorporating the number of animals sampled and the number of positive cases in the model. This adjustment is necessary in order to reduce bias in prevalence estimation due to varying sample size. In the multivariable models, month and year variables were used as fixed effects to model the temporal trend effects. The model used to analyse the FSA data for the respiratory lesions outcome did not converge within 24 hours. A simpler and faster method was used instead, in which a robust cluster function for the unique farm identifier was added to a logistic model. This method provides robust standard errors but does not model the effect of each farm effect separately. This method was then used for all of the respiratory condition models to aid comparison of results. The logistic regression analysis was completed using Stata 12 (StataCorp. 2011).

#### Tail bite lesions

For the tail bite lesions only the BPHS and FSA data were used because this condition is not recorded in FarmFile. As for the respiratory conditions, the number of animals assessed and the number of individual animals that had any positive result for tail bite lesions were summarised per batch. GLMM were used to model the results for tail bite lesions, as described above for respiratory lesions. GLMM analyses were performed with R version 2.12.1 from R Foundation for Statistical Computing. http://www.r_project.org. using the following packages: nortest [17] and lme4 [18].

### Field trials comparison

#### Data description

Four field trials were held in three abattoirs during 2013. These field trials were not part of normal BPHS operations and were carried out especially for the purpose of comparing FSA and BPHS data. Data were recorded at animal level by the BPHS assessors and at batch level by the MHIs, in accordance with their normal practices. During the field trials an external observer was present to guarantee that the same pigs were being scored by BPHS assessors and MHIs. The MHIs were informed about the aim of the field trials before they were carried out and the results of the comparison were discussed with them afterwards. In one abattoir two field trials were carried out at an interval of almost three months.

#### Data management and analyses

Different ways of assessing the same lesions are used, so the data were recategorised into “Conditions” (S3 Table).

The data collected were compared at batch level. A batch was defined as pigs belonging to the same slap mark that were slaughtered in the same abattoir on the same date. For the conditions that were found to have a normal distribution (using the Anderson-Darling test), a Pearson’s correlation test was used to test for correlations between the two proportions (FSA vs BPHS). The conditions that did not follow a normal distribution (right skewed due to the high number of batches with zero prevalence) were categorised according to absence or presence of the condition for each batch. The data were then analysed as binary variables. The Cohen’s Kappa test, which measures the agreement between two methods, was also used. Due to the number of analyses performed in the comparisons (n = 8) any differences were considered statistically significant when P<0.00625, using Bonferroni correction. For the interpretation of the kappa values the following categories, as described by Shoukri and Pause [19] were used: 0 –“poor”, 0.01–0.20 –“slight”, 0.21–0.40 –“fair”, 0.41–0.60 –“moderate”, 0.61–0.80 –“substantial” and 0.81–1.0 –“almost perfect”. The Pearson’s correlation values were interpreted using the following categories, as suggested by Evans [20]: 00–0.19 - “very weak”, 0.20–0.39 - “weak”, 0.40–0.59 - “moderate”, 0.60–0.79 - “strong”,0.80–1.0 - “very strong”.

The data were also reanalysed by comparing the batches with high and low discrepancy, in terms of the number of animals assessed. These two categories were defined as follows:

- Low discrepancy batches: at least 80% of the animals in the batch were assessed by the BPHS assessor, assuming that the MHI assessed the full batch.
- High discrepancy batches: less than 80% of the animals in the batch were assessed by the BPHS assessor, assuming that the MHI assessed the full batch.

This was done because it was assumed that a big difference in the number of animals assessed between these two systems may influence the results. The cut-off of 80% was arbitrarily chosen.

The field trial comparison analyses were performed using the vcd [21] package with R version 2.12.1.

## Results

### Population level comparison

The FSA dataset had the largest number of unique holdings and had the greatest number of pigs assessed compared to the other data sources (Table 1).

**Table 1:** Summary of the three data sources (FSA, BPHS, FarmFile) used to analyse trends.

	FSA	BPHS	FarmFile
Number of pigs assessed	19,534,728	933,771	6,012*
Batches	306,004	21,929	-
Holdings	31,578	2,543	2,699
Identifier	Slap mark, postcode	Slap mark	CPH/ postcode/ unique submission reference
Holdings with postcodes	18,783	2,076	2,076
Number of pigs (%) that could be allocated to regions	17,278,953 (88.5%)	900,840 (96.5%)	5,574 (92.7%)

* Assumed to be a single pig–see Materials and Methods text, Population level comparison–Respiratory lesions, CPH–county parish holding number (identification number for farm or business, which relates to the location of the land).

#### Respiratory conditions

**Fig 1:**
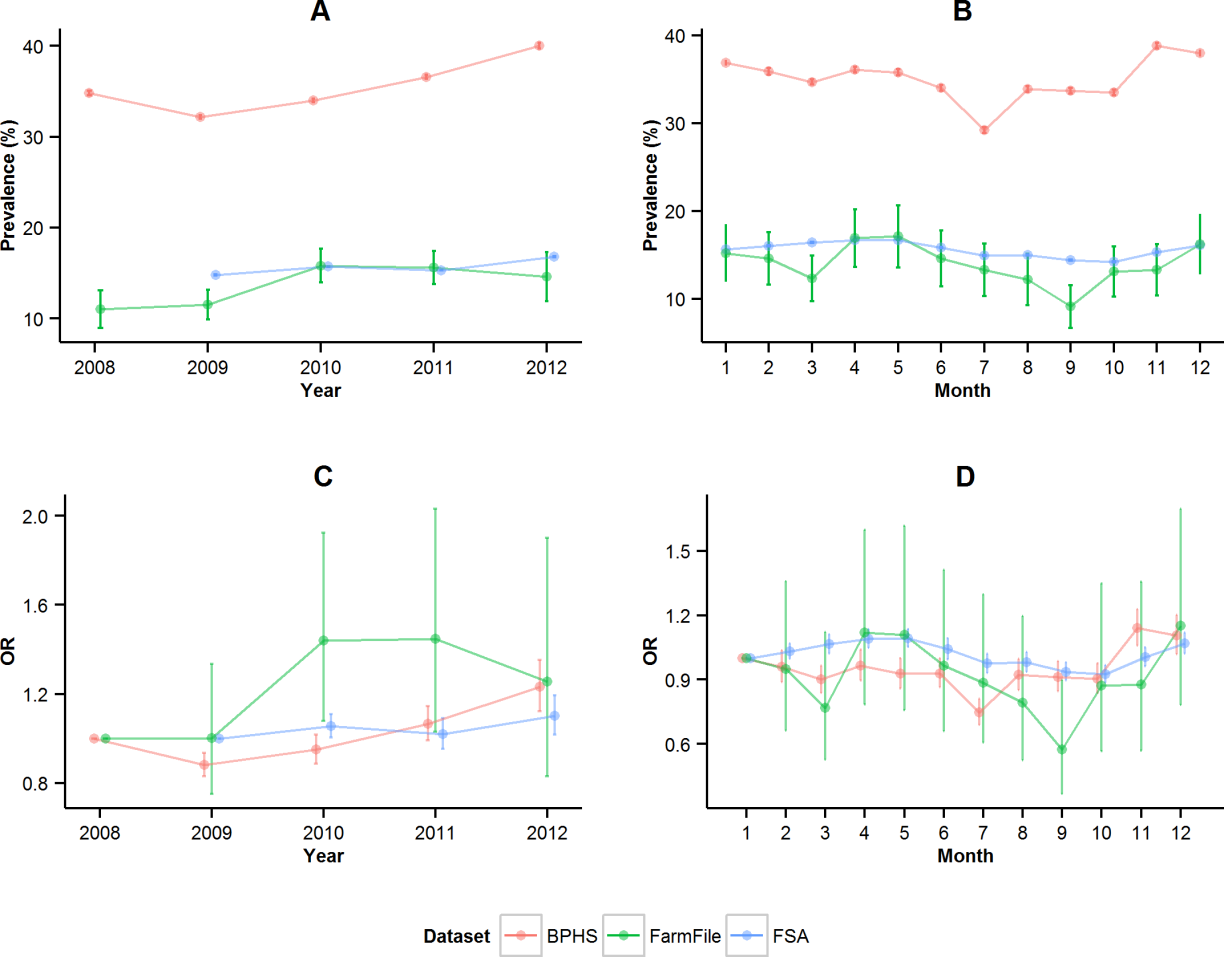
Respiratory disease conditions in the three datasets (FSA, BPHS and FarmFile). Prevalence estimate (dots) and 95% confidence interval (bars) from 2008 to 2012 (A) and from January (1) to December (12) (B). Logistic regression odds ratio (OR) (dots) and 95% confidence interval (bars) from 2008 to 2012 (C) and from January (1) to December (12) (D).

The BPHS data suggested a possible increase in the prevalence of respiratory conditions over the years, with the prevalence of cases rising from 32.2% in 2009 (95%CI: 32.0%-32.4%) to 40.0% in 2012 (95%CI: 39.7%-40.3%) (Fig 1A). The monthly summaries showed that the highest prevalence occurred from November to January, whereas the lowest prevalence occurred in July (Fig 1B). The FSA and FarmFile data also suggested an increased prevalence over the years (Fig 1A). However, the monthly results differed from BPHS. The FSA and FarmFile data had peaks in prevalence occurring in spring and a low prevalence in autumn (Fig 1B). For the FSA data the highest prevalence occurred between March to May, whereas the lowest prevalence occurred in September and October. This was complemented by FarmFile, which had the highest prevalence in April and May and lowest in September (Fig 1B). However, due to the small number of records in this dataset and the overlapping 95% confidence intervals, the monthly prevalence estimates for FarmFile should not be over-interpreted.

In the multivariable temporal analyses, the BPHS model showed significant increasing odds of a pig having respiratory conditions in 2011 and 2012 compared to 2008 (Fig 1C) and that the months July and March had the lowest odds (Fig 1D). The FarmFile model showed that the odds of a respiratory case increased over the years with significant increases in years 2010 and 2011 (Fig 1C). In this model April had the highest odds and September had the lowest (Fig 1D). Finally, the FSA model showed that the odds increased over the years and that 2010 and 2012 were significantly higher than 2009 (Fig 1C). The results for month showed that there were two peaks, with high odds in the spring months and in December (Fig 1D).

**Fig 2:**
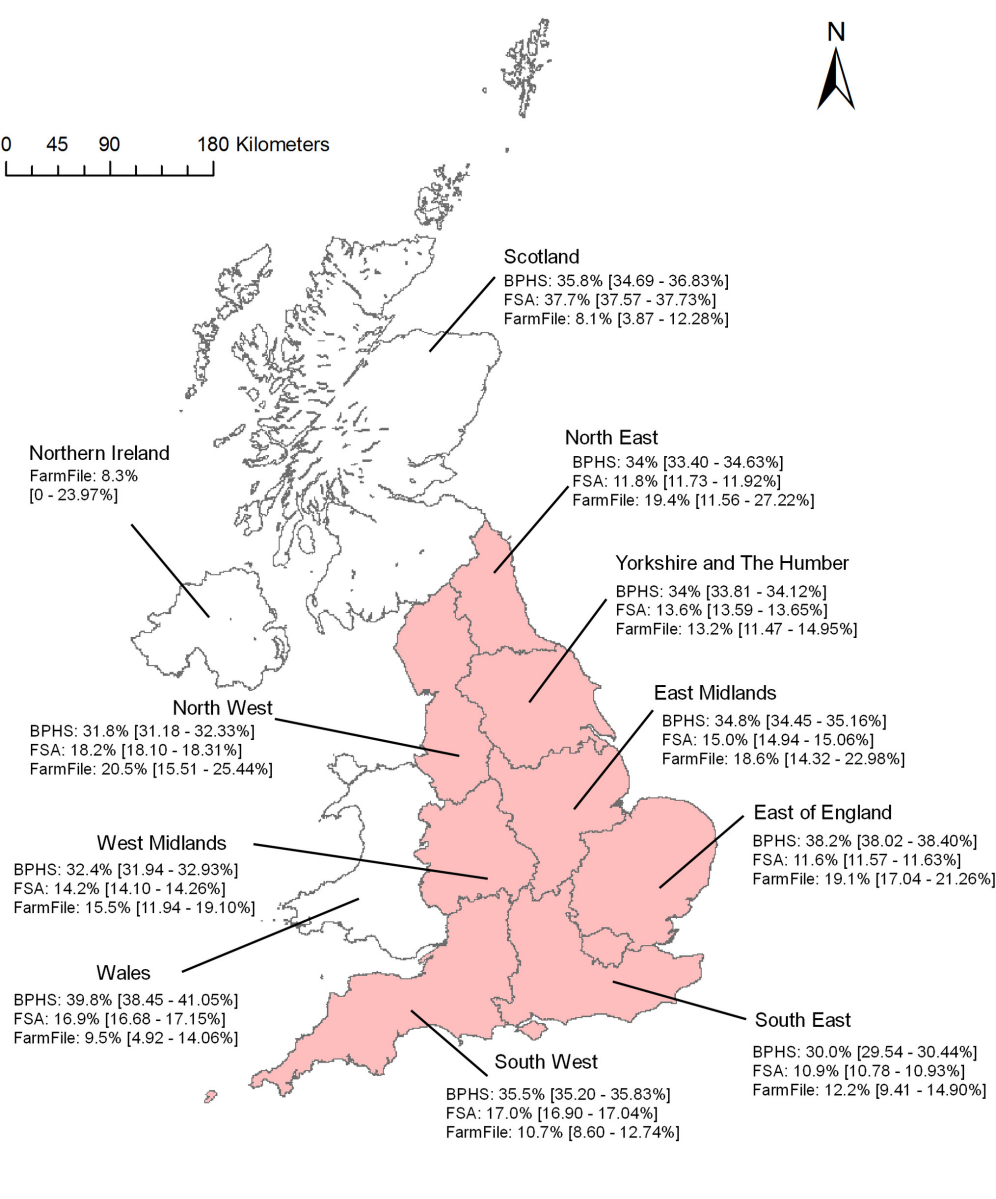
Prevalence of respiratory lesions by UK NUTS1 during the studied period for the three datasets (FSA, BPHS and FarmFile). **England is indicated by the regions shown in pink.** The 95% confidence interval for the prevalence is within brackets.

The prevalence for the BPHS data was comparable across the geographical regions (range 30–39.8%). It was highest in the East of England and Wales, and lowest in South East and North West England (Fig 2). For the FarmFile data, the prevalence was more variable across the regions (8.1–20.5%), being lowest in Scotland and Northern Ireland (where few samples were collected) and highest in the North West and North East. In contrast, for the FSA data, the prevalence was comparable to BPHS data in Scotland, where the prevalence was highest (37.7% [BPHS, 35.8%]) and lower across England and Wales (<20%), being lowest in the South East, North East and East of England (<12%, Fig 2).

#### Tail bite lesions

**Fig 3:**
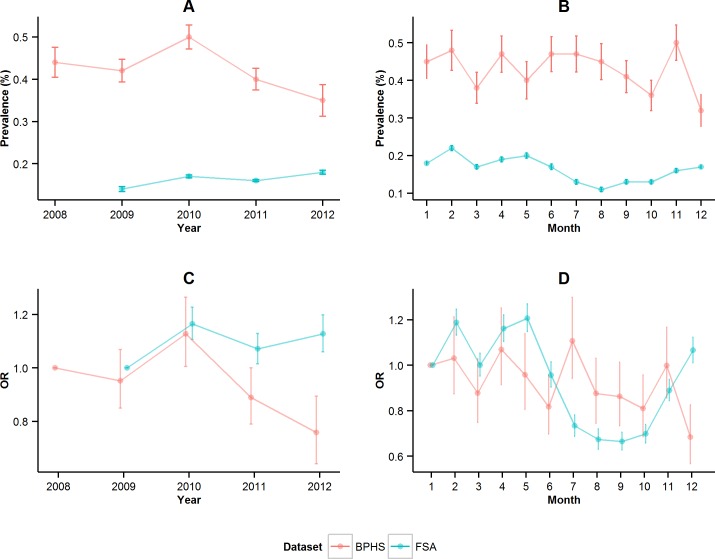
Tail bite lesions in the two datasets (BPHS, FSA). Prevalence estimate (dots) and 95% confidence interval (bars) from 2008 to 2012 (A) and from January (1) to December (12) (B). Generalised Linear Mixed Model odds ratio (OR) (dots) and 95% confidence interval (bars) from 2008 to 2012 (C) and from January (1) to December (12) (D).

The analysis of the BPHS data suggests a general decrease in the prevalence of tail biting from 0.44% in 2008 to 0.35% in 2012. However, year by year comparison shows that the prevalence was highest in 2010 (Fig 3A). There was no clear average monthly pattern (Fig 3B). The FSA data analysis produced contrary results suggesting a general increase in the prevalence from 2009 to 2012 (Fig 3A). The average monthly pattern indicates that the prevalence was highest in February and lowest in August (Fig 3B).

Results from the BPHS data indicate that odds of tial bite lesions declined over the years (Fig 3C). Odds were lower in 2011 and 2012 when compared with 2008. However, the highest odds was in 2010. The odds of tail biting were higher in July and lower in December compared to January (Fig 3D). Temporal patterns differed for FSA: the odds overall the years increased with a similar peak in 2010, however the continued decline in 2012—seen in the BPHS—was not observed. The patterns across the year by month were similar for the first half of the year (January to June) and then differed, with specific differences seen between the datasets in July and December. The odds were higher in May than January and lower in September and August compared to January. However, the 95% confidence intervals for the BPHS dataset are wide and overlap considerably.

**Fig 4:**
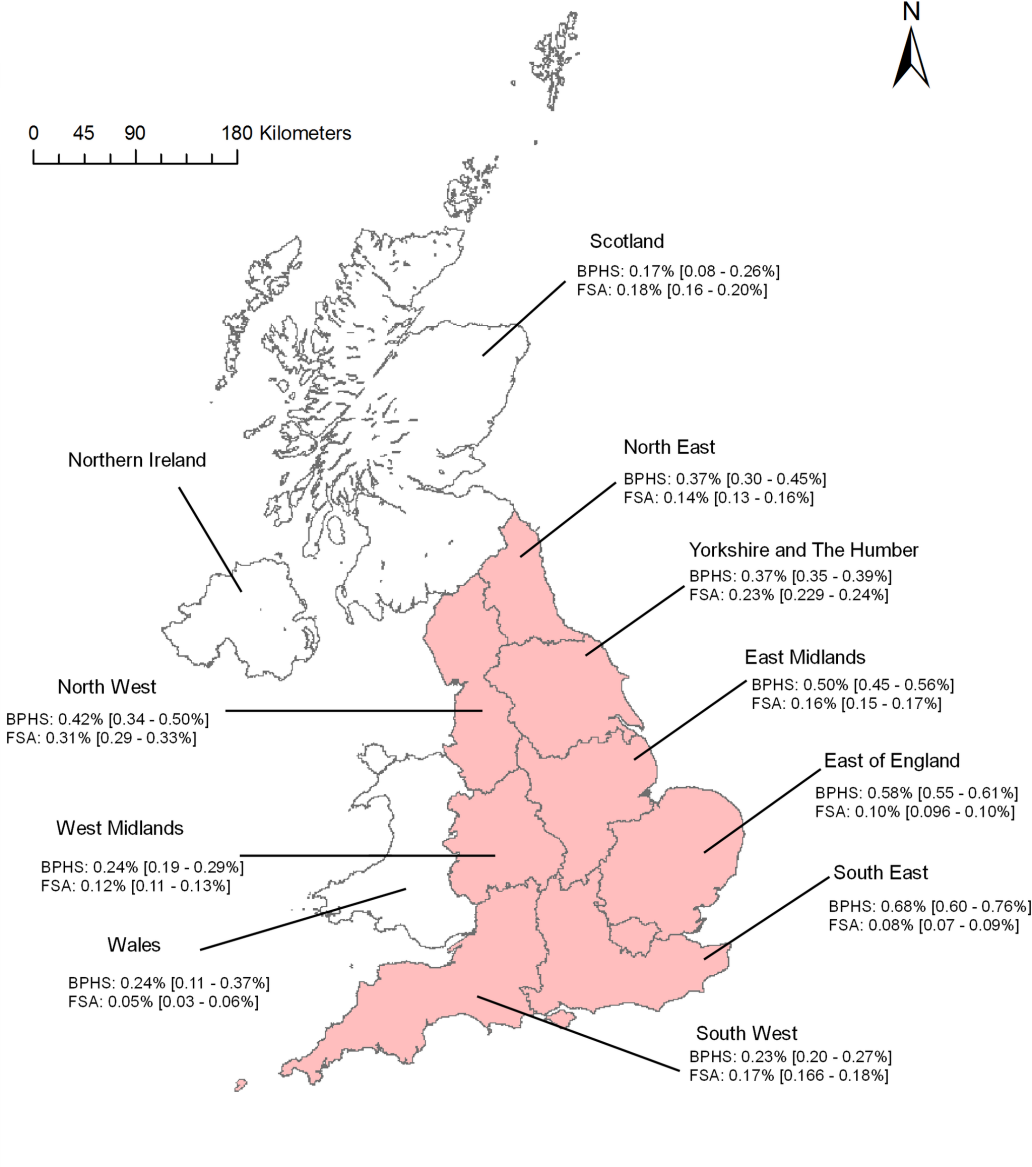
Prevalence of tail bite lesions by UK NUTS1 during the studied period for the two datasets (BPHS and FSA). **England is indicated by the regions shown in pink.** The 95% confidence interval for the prevalence is within brackets.

Descriptive analysis of tail bite lesions by region (Fig 4) using BPHS data suggests that prevalence was highest in the South East and lowest in Scotland (0.68% and 0.17%, respectively). In Scotland it was comparable to the estimate from the FSA data (0.18%). Otherwise the FSA estimates were generally lower than the BPHS ones, with a different distribution; prevalence was highest in the North West (0.31%) and lowest in South East (0.08%) and Wales (0.05%).

### Field trials comparison

Batch level FSA and BPHS data were obtained from a total of 53,479 and 18,748 pigs, respectively, from 332 batches (Table 2). Two batches, from trial 1, were not included in this study due to incorrect recording of the pig slap mark by the MHIs and mixing of conditions data from pigs of different slap marks into the same batch of pigs.

**Table 2:** Dates of the field trials with the number of slap marks, batches and pigs assessed for the three different abattoirs that participated in the field trials for the two data sources (BPHS, FSA).

Abattoirs	Trial number	Dates (2013)	Number of slap marks	Number of batches	Number of pigs assessed
A	1: week	22 to 26/04	35	50	2,479 (BPHS), 8,805 (FSA)
	1: days	21/02, 8/05, 13/05, 14/05	23	34	1,685 (BPHS), 5,895 (FSA)
	3: week	8 to 12/07	46	56	3,374 (BPHS), 12,729 (FSA)
B	2: week	17 to 21/06	87	101	4,408 (BPHS), 13,104 (FSA)
	2: days	28/06, 2/07, 8/07	46	49	3,389 (BPHS), 8,249 (FSA)
C	4: week	30/09 to 4/10	22	24	2,044 (BPHS), 2,765 (FSA)
	4: days	7/10, 18/10, 22/10	17	18	1,369 (BPHS), 1,932 (FSA)

The overall results show a moderate correlation between the inspection methods for pericarditis and pleurisy and a strong correlation for pneumonia. The agreement is moderate for milk spots and slight for tail bite, peritonitis and pyaemia (Table 3). The results at abattoir C (which had a low line speed) showed better agreement for milk spots, pyaemia and abscesses in the lungs compared to the other abattoirs although not statistically significant (Table 3). Abattoir C also had a moderate correlation for pericarditis and a moderate to strong correlation for pneumonia (Table 3).

**Table 3:** Summary of the correlation/agreement results between the two datasets (BPHS, FSA) for pericarditis, pleurisy, pneumonia, milk spots, tail bite, pyaemia, peritonitis and abscesses in the lung for each of the week field trials and all data of the field trials, with significance levels.

Condition	Abattoir A: Week trial 1	Abattoir B: Week trial 2	Abattoir A: Week trial 3	Abattoir C: Week trial 4	All data (week plus days trials)
Pearson’s correlation coefficients					
Pericarditis	0.511***	0.612***	0.304*	0.531**	0.415***
Pleurisy	0.162	0.545***	0.507***	0.393	0.473***
Pneumonia—v1	0.837***	0.571***	0.684***	0.769***	0.650***
Pneumonia—v2	0.883***	0.572***	0.798**	0.622**	0.687***
Pneumonia—v3	0.826***	0.597***	0.746***	0.405*	0.653***
Cohen’s kappa agreement values					
Milk spots	0.32	0.36	0.325**	0.408	0.414**
Tail bite	0.14**	0.10***	0***	0.023***	0.038***
Pyaemia	0.23*	0	0***	0.333	0.069***
Peritonitis	0.33*	0.18	0.062***	0.167**	0.175***
Abscesses in the lung	0.18	0.19***	0.103***	0.250*	0.131

*p<0.05

** p<0.01

*** p<0.001

Pneumonia–v1: number of animals with enzootic pneumonia-like lesions score >0, or/and Viral-like pneumonia or/and pleuropneumonia lesions; Pneumonia–v2: number of animals with enzootic pneumonia-like lesions score >5, or/and Viral-like pneumonia or/and pleuropneumonia lesions; Pneumonia–v3: number of animals with enzootic pneumonia-like lesions score >10, or/and Viral-like pneumonia or/and pleuropneumonia lesions

When batches with low or high discrepancy in the number of pigs assessed were compared (Table 4), the correlation for pericarditis was higher for the batches with low discrepancy compared to the batches with a high discrepancy. For pneumonia the opposite was observed and no major difference was seen for pleurisy (Table 4). In general, the agreement for peritonitis was better for the batches with low discrepancy compared to the ones with high discrepancy (Table 4).

**Table 4:** Summary of the correlation/agreement results between the two datasets (BPHS, FSA) for pericarditis, pleurisy, pneumonia, milk spots, tail bite, pyaemia, peritonitis and abscesses in the lung for low and high discrepancy batches, with significance levels.

Condition	Low discrepancy	High discrepancy
Pearson’s correlation coefficients		
Pericarditis	0.702***	0.364***
Pleurisy	0.488**	0.467***
Pneumonia–v1	0.483**	0.676***
Cohen’s kappa agreement values		
Milk spots	0.648	0.372**
Tail bite	NC	0.097***
Pyaemia	NC	0.091***
Peritonitis	0.325**	0.172***
Abscesses in the lung	0.155	0.139

Low discrepancy batches: at least 80% of the animals in the batch were assessed by the BPHS assessor, assuming that the MHI assessed the full batch, high discrepancy batches: less than 80% of the animals in the batch were assessed by the BPHS assessor, assuming that the MHI assessed the full batch

** p<0.01

*** p<0.001

NC: Not possible to estimate due to zero results in both positive categories, Pneumonia–v1: number of animals with enzootic pneumonia-like lesions score >0, or/and Viral-like pneumonia or/and pleuropneumonia lesions.

## Discussion

The aim of this study was to investigate if pig abattoir inspection data could be used for surveillance purposes. To achieve this we compared the prevalence (over the time period and regions) of two conditions at population level and investigated the agreement at batch level between FSA and BPHS data.

### Population level comparison

The major difference between data sources was in the values of the prevalence estimates generated. These were higher for respiratory lesions in BPHS in comparison to both FSA and FarmFile, in which the estimates were similar. In addition, even though the prevalence of tail bite lesions is approximately 100 times less, the estimates were equivalently higher in the BPHS dataset compared to the FSA dataset. While some difference would be expected due to the differences in the populations sampled and in the criteria for inclusion in the respiratory lesion category of each dataset, this consistent difference between the FSA and BPHS dataset, even when the condition is classified in a similar way (tail bite lesions), suggests that something else is happening. A similar finding was observed in another study that compared routine abattoir inspection findings with systematic health monitoring in pigs in Denmark [22]. This seems to suggest that there are differences in recording sensitivities between the two systems i.e. the BPHS assessors detect a higher proportion of carcasses affected with respiratory conditions compared to the MHIs; similarly with a low prevalence condition such as tail bite lesions. The FarmFile data is a very different subset of the population, relating to more overtly diseased animals and diagnostic requests (a minority of which were for monitoring purposes). Why this should have similar prevalence estimate values for respiratory lesions to those in the FSA dataset is not entirely clear, unless it is related to the sensitivity of diagnostic uncertainty.

For respiratory conditions the yearly and monthly patterns were similar between the different data sources. For tail bite lesions the two data sources showed differences in both temporal patterns. The major difference was a year effect in 2012 and variations in the second half (June–December) of the year. In both sets of analyses the patterns in the FSA data are smoother. This would be expected from a larger, continuously collected dataset that encompasses the wider population. The FSA and FarmFile data complement each other for respiratory conditions, which was expected; as subclinical conditions detected in the wider population may either be an indicative lead into an increase in clinical observations–and subsequent submission for diagnosis–or, as a sequela. Similarly one would expect the FarmFile and BPHS data to complement each other (clinical v. subclinical) but for both to be more variable than the FSA data, as they are from smaller but potentially more investigatory minded systems than the general abattoir inspection. BPHS is known to cover a smaller population of farms, with members representing only 75% of the commercial units [23]. Furthermore, the intermittent (voluntary and quarterly) nature of the data collection will have an effect on the variability in prevalence estimates, particularly on a monthly basis. All of these aspects may contribute to the differences observed between the BPHS and FSA data for tail bite lesions. Additionally, the age of the assessed pigs in the datasets differs. BPHS pigs are slaughter age, finished pigs with an average age of 5.7 months [24], as are the vast majority of those contributing to the FSA dataset. FarmFile, however, can include data from pigs of any age, although those pigs being submitted for respiratory disease are usually younger than typical slaughter age pigs. This would have the consequence that, in the temporal analysis, an increase in prevalence of respiratory disease detected in weaners or growers, recorded by FarmFile, might not be expected to be reflected in lesions observed at slaughter until 6–16 weeks later i.e. the FSA and BPHS data would be expected to ‘lag’ FarmFile

The spatial differences between the datasets reflect the overall prevalence difference, with BPHS regional estimates being consistently higher than the FSA estimates for both lesions in all regions, with the noticeable exception of Scotland. Here the BPHS and FSA prevalence estimates concur, however the number of Scottish farms that send pigs to slaughter in England is very small and not representative of the Scottish situation. For each of the two conditions, the variation in the regional spatial distribution within each dataset is not the same. It is not possible, therefore, to tell whether there is true regional variation in the conditions themselves without considerable further investigatory work into other potential contributory factors. For example, regional differences due to assessor differences should be minimised in the BPHS dataset as standardisation exercises are held, whereas these are not common practice between MHIs and abattoirs.

There are other data limitations that might influence the results. Firstly, BPHS and FSA record non-specific lesions, which has the advantage of high sensitivity but low specificity. In contrast, FarmFile records specific diagnoses with high specificity but low sensitivity due to submission bias. Secondly, the data, even within recategorised conditions, are not directly comparable due to the different definitions and criteria for recording (e.g. combination of multiple respiratory conditions in FarmFile data). This has resulted in a loss of sensitivity for patterns of specific respiratory conditions. Thirdly, there is also potential for misclassification; in the FarmFile data the main presenting condition would have been recorded, but in some cases other secondary conditions were not recorded. This is a known hazard in the FSA inspection system, too [13]. In addition, for the FSA data, the conditions details were summarised into the total number of animals that had body parts rejected due to a condition. As data on individual animals was not provided, it was possible that some double counting of animals with conditions was included e.g. a batch with one record of tail bite and a record of fight/bite may have been the same animal and would have been recorded as two animals instead of one. An improvement, for future analyses of FarmFile data, would be to analyse only diagnostic samples for which respiratory clinical signs had been noted by the submitting veterinary practice. A further refinement would be to split carcass and non-carcass diagnostic submissions, as carcass submissions would be indicators of more severe incidents of respiratory conditions.

### Field trial comparison

The BPHS and FSA data were also compared in terms of agreement per batch. Overall, the prevalence of respiratory conditions was higher within BPHS data than the FSA (data not shown) and the results of the field trials showed a moderate correlation between data sources for pericarditis and pleurisy and a strong correlation for pneumonia. This is similar to findings by Nielsen and colleagues [22], except for pleurisy where the correlation was stronger than the one observed in this study. The agreement between data sources was moderate for milk spots and slight for tail bite, peritonitis and pyaemia. For some conditions (pericarditis and peritonitis) with low prevalence, the agreement between data sources improved when all, or almost all, animals in the batch were assessed and not only a sample of the batch. This suggests that assessing a subset of animals per batch (as BPHS currently does) is not enough to account for the variability associated with low prevalence conditions (e.g. for detecting a disease with 1% prevalence in a group of 100 animals with 95% CI, 95 animals should be tested) [25].

One limitation of this comparison was the power of the statistical analysis of the field trials. This was low because only three abattoirs were involved, with a low number of batches assessed. However, it was a resource intensive exercise and the abattoirs were chosen to represent the situation of English abattoirs (e.g. abattoir A and B were high output abattoirs while abattoir C was a low output abattoir and therefore had a different line speed). The MHIs were aware of the aim of the field trials before they were carried out, as were the BPHS assessors, and the results were discussed with all involved in the field trials at the end of each day. This potentially may have affected the outcome of the field trials. While a blinded process would have been preferable that was not possible and both sets of assessors had the opportunity to raise their game on the day. One abattoir (A) had two field trials. This meant that all involved in the trials in that abattoir were aware, in the second field trial, of previous pitfalls identified and therefore could have changed their procedures. However, an improvement in agreement for the different conditions assessed from week 1 to week 3 for abattoir A was not observed. The comparison between outcomes is not one between equivalent inspections; it was a comparison between two types of inspection.

Another potential limitation of abattoir inspection data could be its differing ability to detect conditions. The sensitivity (probability of detecting conditions actually present in the diseased group) and specificity (probability of correctly identifying healthy carcasses in a healthy group) of routine abattoir inspection for parasitic, intestinal and heart disorders was considered low in a study that compared two groups of observers (regular meat inspectors and two veterinary researchers) [26]. In addition, in a recent Danish study [22], pericarditis, pleuritic and lung lesions data collected through routine abattoir inspection were compared to data collected through a systematic health monitoring system (voluntary scheme for farmers having clinical problems) for the same batches. The results suggested that the correlation was moderate for pleuritic and lung lesions, but poor for pericarditis and the authors concluded that caution should be used whenever routine abattoir inspection data are used for purposes other than those for which they were originally intended.

### Potential for animal health surveillance purposes

The results of our work suggest that the FSA data could, potentially, be used to measure the apparent prevalence of conditions and monitor trends over time at the population level. This holds, as long as the system remains stable in denominator population (number and type of pigs slaughtered; management types, ages, etc.), methodology and application. This, however, is not the case: the current FSA system has been subjected to challenges, such as the transition to visual inspection from June 2015 and the retraining of MHIs. These changes will have affected the types of conditions that can be recorded and could affect the performance of the system over time, respectively. Neither aspect was evaluated in this study. Nielsen and colleagues [22] have suggested that, at least for pericarditis, some cases will be missed at visual inspection, which can result in a loss of overall sensitivity. The industry would also need to accept that the prevalence in this population is different than the estimates acquired via the BPHS because the two inspection systems are different.

While useful for temporal trends, further work would be required to investigate whether there is the potential to detect emerging or re-emerging diseases. The continuous nature of the data collection and the large numbers inspected make it easier to detect a statistically significant change in the overall population in a timely manner. The loss of sensitivity for some conditions and the larger scale may, however, mask more localised fluctuations that are of clinical significance within specified populations or locations. Alternatively, it could draw a number of these fluctuations together into a coherent indication of a problem that would not otherwise be recognised. Furthermore, it is expected that emerging and re-emerging endemic diseases will usually be detected before slaughter; for example by production and clinical monitoring at farm-level. Abattoir inspection could act as an ultimate ‘fail-safe’ in the case of failure at this level, particularly when faced with an insidious, sub-clinical, situation. It enables an alarm to be raised when the other components fail [5,27]. Elsewhere, several studies [4,5,6,7,28,29,30,31] have evaluated the use of abattoir data for integration in syndromic surveillance systems, for the early detection of emerging diseases in livestock animals or to measure animal health and welfare. Some of the advantages identified were the good coverage and availability of syndromic indicators; while some of the limitations were the lag of time between reporting and occurrence of disease in live animals and the influence on the results of abattoir-related factors.

The potential utility of the FSA data for the provision of evidence to inform decision making and individual action at the producer level (i.e. could FSA data replace BPHS data?) is hampered by a number of issues; this is illustrated by the field trial batch level results. The major issues are the recording of correct slap mark for each pig and the double counting of animals with multiple conditions. Standardisation of MHIs in terms of assessment and recording of conditions is also required to give confidence to producers and their veterinarians. The lack of agreement between the two systems and the higher prevalence of lesions recorded in the BPHS system (especially for pneumonia and pleurisy) may suggest that, for the measurement and monitoring of endemic disease and welfare conditions, the detection of change and the evaluation of the effects of any control measures that are implemented at producer level, the BPHS system performs better than the FSA currently could. This probably occurs because BPHS assessors are focused only on a limited number of standardised specific animal health related lesions, while FSA ante and post mortem inspection has the wider primary remit to protect public health. However, given the reduced ability to detect conditions with low prevalence in large batches and the quarterly nature of the BPHS inspection, it may be less suited than the FSA data to the early detection of emerging or re-emerging diseases, at a producer level.

The data sources investigated in this paper are considered to be three of the most robust data sources currently available for the British pig industry. The analyses highlighted that no single source could fulfil all the purposes that a surveillance system may be asked to meet; however, they each have useful contributions to make as separate components of an animal health surveillance system. As each of the data sources differs in terms of coverage and representativeness of the pig industry, amalgamating all the data together could be attempted, as could integration through statistical analyses. Such approaches were not within the remit of this study. The FSA data could be interpreted in parallel with FarmFile and BPHS data, or with FarmFile data alone to provide monitoring of trends of the chosen indicators at population (i.e. national) level. Stärk and colleagues [5] drew a similar conclusion that the combination of abattoir inspection with other surveillance components can provide more information than abattoir inspection alone. The FSA data requires improvement to be effective for use at the producer level. Recently AHDB–Pork began working with the FSA and abattoirs to improve the FSA system. The aim is to bring it to a position where it can provide similar information on pig health and welfare issues from post mortem data, to that which is currently delivered through the BPHS. When such a system is in place further comparisons should be done.

In this paper, the abattoir inspection data for the pig industry could be assessed because of the existence of the purposively designed abattoir based health scheme data. For the other livestock industries in Great Britain no such comparative schemes exist. However, it is likely that the same conclusions can be made: the FSA data can be interpreted in parallel with FarmFile to provide monitoring of trends of any chosen indicators at population (i.e. national) level but requires improvement to be valued at producer level. Standardised collection and recording of the core data requirements, including those variables that enable linkages to be made with other data sources (slap mark, CPH and postcode) is vital. This and the provision of a background standardised description and evaluation of the data sources is essential for any attempt to use multiple data sources for surveillance purposes. Similar conclusions may apply to other countries where statutory abattoir inspection data are collected; especially within the EU, as data collection is regulated by the same legislation. It would, however, require an evaluation of the inspection and data collection processes before robust comparisons can be drawn between countries.

## Conclusion

There is potential to use abattoir inspection data, as a component of an animal health and welfare surveillance system, in order to monitor temporal trends and regional differences of chosen indicators at population (i.e. national) level. Further work is required to evaluate its potential for the early detection of emerging or re-emerging diseases at a population level; however, there is the potential to do this with low prevalence conditions at producer level (i.e. within herd/batch). To inform decision making at producer level, the lack of agreement is greater and therefore improvements need to be made if the FSA system is to replace the BPHS system.

## Supporting Information

S1 Table Description of characteristics of the three data sources (FSA, BPHS, FarmFile) for pigs in Great Britain. (DOCX)

S2 Table The information within each dataset (FSA, BPHS and FarmFile) and how it was recategorised into ‘conditions’ for use in the population-level analysis. (DOCX)

S3 Table The information recorded by each set of assessors/inspectors and how it was recategorised into ‘conditions’ for use in the batch-level comparison. (DOCX)
