# Supplementary material for: Pig Abattoir Inspection Data: Can It Be Used for Surveillance Purposes?
Source: PLoS One. 2016 Aug 26;11(8):e0161990. doi: 10.1371/journal.pone.0161990 (PMC5001630; doi:10.1371/journal.pone.0161990)
Supplement: S1 Table — (DOCX) [file pone.0161990.s001.docx]

S1 Table: Description of characteristics of the three data sources (FSA, BPHS, FarmFile) for pigs in Great Britain.

| Characteristics | Data sources | | |
| --- | --- | --- | --- |
| Name of the system | FSA abattoir inspection  (also known as Collection and Communication of Inspection Results (CCIR) system) | British Pig Health Scheme (BPHS) | FarmFile |
| Name of the agency | Food Standards Agency (FSA) | Agricultural and Horticultural Development Board (AHDB) - Pork | Animal and Plant Health Agency (APHA) |
| Recording system | The MHI record all conditions observed either on a “touch screen”, or on paper, depending on the system implemented in each abattoir. This information is then transferred to the abattoir records and FSA and is printable for each batch (identified by the individual slap mark). | Data are recorded at the abattoir from inspections of 50 pigs from a batch of slaughtered pigs by assessing every other pig on the slaughter line. The detailed operation of BPHS has been described previously [23] | Records information from voluntary laboratory submissions to the Great Britain APHA network. Carcass and non-carcass submissions are submitted through private veterinary surgeons, to the network, for laboratory testing and diagnostic investigation. |
| Type of data | Ante and post-mortem inspection as required by the legislation (Regulation (EC) 854/2004). Presence or absence of specific conditions that lead to rejections (partial or total). For each batch of pigs slaughtered the number of pigs in the batch, the type of pigs, and the number of conditions observed is recorded. Finishing pigs and culled sows and boars | Presence/absence or severity of 12 different macroscopic conditions detected in slaughtered pigs (in the pluck, offal and on the skin). Finishing pigs data. | Each submission is given a specific code that indicates the diagnosis(es) obtained. Strict diagnostic criteria are followed for endemic diseases of farmed livestock and wildlife. Cases where no diagnosis is reached (DNR) are also coded and reviewed on a quarterly basis as a means of detecting possible undiagnosed emerging disease. Pigs of all ages but predominantly young pigs. |
| Type of system | Compulsory | Voluntary (only members) | Voluntary (voluntary submissions to the laboratories by private veterinary surgeons) |
| Coverage | All pigs slaughtered in English abattoirs (including batches of Scottish pigs sent to slaughter in England but not representative of this population). | Batches of pigs from scheme members and non-members slaughtered in English abattoirs in the specific assessment days (including batches of Scottish pigs sent to slaughter in England but not representative of this population). | England and Wales predominantly but also receives submissions from Scotland and Northern Ireland (although not representative of these countries). |
| Advantages | Increased coverage using an existing data collection system. Data are available on many health and welfare conditions (including some exotic disease conditions) | Standardisation of the assessors. Targeted to specific endemic conditions.  Improved sensitivity of detection. | High specificity. Standardised case definitions. Rapid dissemination and in-depth investigation of new syndromes [8] |
| Limitations | Lack of sensitivity. Lack of standardisation with multiple operators and conditions. Poor data quality. Coverage of only healthy pigs sent to slaughter [13] | Specificity of the case definitions is limited.  Voluntary nature of participation (good coverage for large producers but not for small producers). Only suitable for health problems leading to lesions detectable at slaughter [8]. Quarterly assessments only. | Biased subset of the livestock population (diseased animals). Highly dependent on the submission rate [8]. |
