# Supplementary material for: Pig Abattoir Inspection Data: Can It Be Used for Surveillance Purposes?
Source: PLoS One. 2016 Aug 26;11(8):e0161990. doi: 10.1371/journal.pone.0161990 (PMC5001630; doi:10.1371/journal.pone.0161990)
Supplement: S2 Table — (DOCX) [file pone.0161990.s002.docx]

S2 Table: The information within each dataset (FSA, BPHS and FarmFile) and how it was recategorised into ‘conditions’ for use in the population-level analysis.

| **Lesions/**  **diseases** | **FSA** | **BPHS** | **FarmFile** |
| --- | --- | --- | --- |
| Respiratory | Conditions:  (before May 2012)  Abnormal Breathing Rate/Depth (AM)  Coughing (AM)  Respiratory (AM)  Rhinitis (AM)  Pleurisy (PM - C)  TB-like lesions (PM - C)  (after May 2012)  Abnormal respiratory signs (AM)  Pleurisy (PM -C)  TB-like lesions (PM - C)  Pleurisy (PM - O)  Pneumonia with abscess (PM - O)  Pneumonia without abscess (PM -O)  Pneumonia with pleurisy (PM -O)  TB-like lesions (PM – O)  Generalised tuberculosis (suspect) (PM – G)  Septic Peritonitis and Pleurisy (PM – G)  Septic Pleurisy (PM – G) | Conditions:  EP-like lesions,  Viral-like lung lesions, Acute pleuropneumonia, Chronic pleuropneumonia  Pleurisy | Pneumonia due to Pasteurella multocida,  PCVD - pneumonia,  Pneumonia associated with PRRS,  Pleurisy not specified,  Pneumonia not specified,  Parasitic pneumonia,  Swine flu,  Inclusion body rhinitis,  Progressive atrophic rhinitis due to toxigenic Pasteurella multocida,  Pneumonia due to Haemophilus parasuis,  Pneumonia due to Actinobacillus pleuropneumonia,  Pneumonia due to Mycoplasma hyopneumoniae,  Pneumonia due to Bordetella bronchiseptica,  Pneumonia due to Mycoplasma hyorhinis,  Diagnosis not listed - respiratory disease,  Diagnosis not reached - respiratory disease was included in our definition.  Also included PRRS and Glassers where respiratory conditions were also indicated with the diagnosis |
| Tail damage | Conditions:  Tail bite (PM) | Conditions:  Tail bite | Not recorded |

Legend: Ante mortem (AM), post mortem (PM), carcass (C), offal (O), generalised conditions (G), tuberculosis (TB), enzootic pneumonia (EP), porcine reproductive and respiratory syndrome (PRRS), porcine circoviral disease (PCVD).
