# Supplementary material for: Pig Abattoir Inspection Data: Can It Be Used for Surveillance Purposes?
Source: PLoS One. 2016 Aug 26;11(8):e0161990. doi: 10.1371/journal.pone.0161990 (PMC5001630; doi:10.1371/journal.pone.0161990)
Supplement: S3 Table — (DOCX) [file pone.0161990.s003.docx]

S3 Table: The information recorded by each set of assessors/inspectors and how it was recategorised into ‘conditions’ for use in the batch-level comparison.

|  | **Data sources** | |
| --- | --- | --- |
| **Conditions** | **BPHS** | **FSA** |
| Abscess in the lung | Number of individual animals that had any positive result for the condition. | Total number of animals with abscesses in the lung, with pneumonia with abscesses in the lungs or in the full pluck. |
| Milk spots | Number of individual animals that had any positive result for the condition. | Total number of animals with milk spots in the liver (localised or generalised). |
| Pericarditis | Number of individual animals that had any positive result for the condition. | Total number of animals with pericarditis in the heart. |
| Tail bite | Number of individual animals that had any positive result for the condition. | Total number of animals with tail bite on the carcass. |
| Peritonitis | Number of individual animals that had any positive result for the condition. | Total number of animals with peritonitis in:  -the guts, pancreas, spleen or liver  - the pelvis and trim (carcass)  -septic peritonitis and pleurisy |
| Pleurisy | Number of individual animals that had any positive result (mild or severe) for the condition. | Total number of animals with pleurisy in:  - The lungs and pluck (including pleurisy and pneumonia)  - the carcas (pleura and trim)  -septic pleurisy and/or septic peritonitis and pleurisy |
| Pneumonia | Pneumonia – v1:  Number of animals with enzootic pneumonia-like lesions score >0, or/and Viral-like pneumonia or/and pleuropneumonia lesions  Pneumonia – v2:  Number of animals with enzootic pneumonia-like lesions score >5, or/and Viral-like pneumonia or/and pleuropneumonia lesions  Pneumonia – v3:  Number of animals with enzootic pneumonia-like lesions score >10, or/and Viral-like pneumonia or/and pleuropneumonia lesions | Total number of animals with pneumonia with and without abscesses, pneumonia and pleurisy in:  - the lungs,  - the full pluck. |
| Pyaemia | Number of individual animals that had any positive result for the condition. | Total number of animals with suspect pyaemia/ multiple abscesses. |
